# On-demand treatment of icatibant in a patient of hereditary angioedema with KNG1 mutation

**DOI:** 10.1016/j.gendis.2026.102233

**Published:** 2026-05-14

**Authors:** Wo Yao, Boyun Yang, Huiying Wang

**Affiliations:** Department of Allergy and Clinical Immunology, The Second Affiliated Hospital of Zhejiang University School of Medicine, Hangzhou, Zhejiang 310009, China

Hereditary angioedema (HAE) is a rare genetic disease characterized by recurrent cutaneous and submucosal swelling of multiple organs.^1^ A deficiency or defect of C1 esterase inhibitor (INH) encoded by the SERPING1 gene, leading to the excessive release of bradykinin, is considered the main mechanism. Icatibant, a specific and selective competitive antagonist of the bradykinin B2 receptor, is recommended for the on-demand treatment of HAE with comparable efficacy/safety supported by the clinical trials and real-world observations.^2^

Recently, HAE with normal C1–INH (HAEnC1) has been increasingly recognized. It presents with similar symptoms but arises from a distinct genetic variant. Therapeutic strategies remain a big challenge due to limited clinical trial data. We report a case of HAEnC1 with a novel mutant of the KNG1 gene who achieves fast and effective relief from an acute attack after on-demand treatment with icatibant, trying to raise awareness of the disease and add more clinical experience.

## Case report

**Figure 1:**
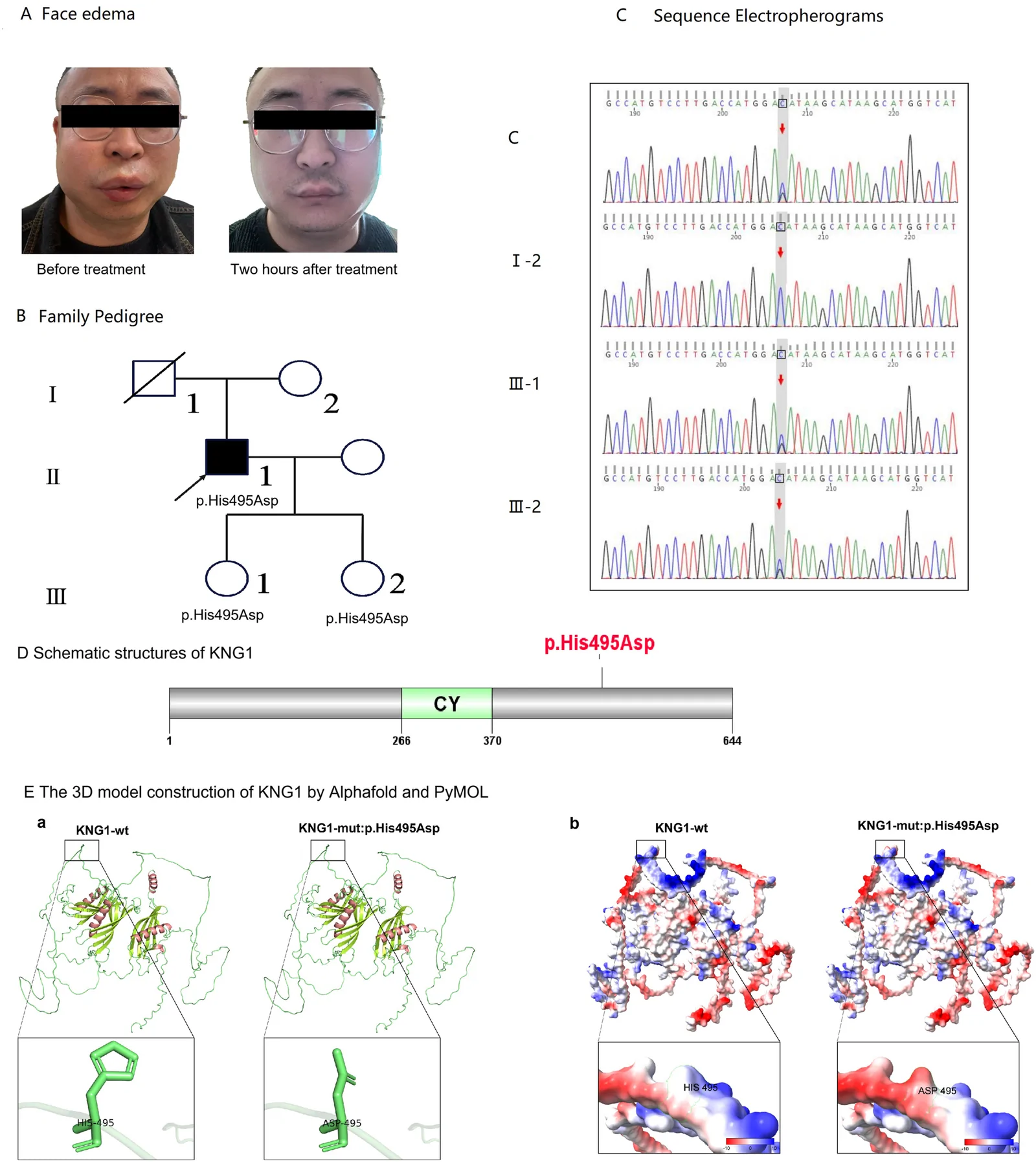
Clinical symptoms and genetic study of the case investigated. **(A)** Face edema on admission and 2 h after icatibant treatment. **(B)** Family tree. A heterozygous mutation of *p*.*His495Asp* in the proband and his two daughters. **(C)** Sanger sequencing revealed a heterozygous c.1483C>G variant in the KNG1 gene, shared by the proband and his two daughters. **(D)** Schematic structures of *KNG1*. The novel variant *p*.*His495Asp* is shown in red. **(E)** The 3D model construction of *KNG1* by Alphafold and PyMOL. The substitution of a *histamine* for an *aspartate* did not change the hydrogen bond around and the tertiary structure of the protein **(a)**. The protein electrostatic properties of residue 495 change from neutral to negative **(b)**.

A man in his 40s was admitted to the emergency department for facial edema and tightness of the throat for 3 h (Fig. 1A). He had recurrent swelling of the face and extremities for 4 years with the involvement of the scrotum. Abdominal pain and laryngeal obstruction were not previously confronted. The attack presented as isolated edema without skin wheals or itching; the frequency was about two to three times a month. Each episode attacked only one or two sites at most. Oral anti-histamines such as loratadine, Zytec, etc. were taken for relief but without response, and the symptoms would mitigate within 2–3 days naturally. The smell of molds or bedding could be the trigger. He had a history of hypertension controlled evenly with felodipine, a calcium channel blocker. Based on his classical manifestations, HAE was suspected, and 30 mg icatibant was injected subcutaneously empirically. The edema subsided quickly within 2 h, and he breathed freely (Fig. 1A).

Laboratory examination revealed a higher serum C4 level at 497 mg/L (normal range, 100–400), antigenic C1–INH at 0.37 g/L (normal range, 0.21–0.39), and the activity of C1–INH at 93% (normal range > 68%).

He denied the family history of HAE. His mother and two daughters did not have the experience of edema, while details of his deceased father were unclear (Fig. 1B). Whole-exome sequencing identified a heterogeneous variant of the *KNG1* gene on exon 12 (chr3: 186459668, NM_001102416.3: c.1483 C>G), predicting the amino acid changes of p.*His495Asp*. This is a missense mutant and not documented in the gnomAD database. Further family genotyping revealed that his two daughters inherited this variant (Fig. 1C). Protein modeling and functional prediction using the software of Alphafold and PyMOL demonstrated that the mutation did not impact the tertiary structure of the protein but might lead to the instability of the protein (Fig. 1D and E).

## Discussion

HAEnC1 is extremely rare, and a genetic test is needed for a proven diagnosis. Based on the recommendation of the United States HAE Association Medical Advisory Board, our patient is diagnosed as HAEnC1 with typical clinical manifestations and identification of the mutation of the KNG1 gene. Our patient showed recurrent isolated skin edema involving multiple systems, and spontaneous remission, but no response to antihistamine reagents. This excluded histamine-mediated angioedema and suspected bradykinin-mediated angioedema. The medication for his hypertension further excluded angiotensin-converting enzyme (ACE) inhibitor-induced angioedema. Identification of the mutation of the KNG1 gene further confirmed this diagnosis. The reports of HAE caused by *KNG1* mutation are very limited. Bork K first reported a novel variant of c.1136T>A in exon 10 of the KNG1 gene in a German family in 2019, which changes the N-terminal cleavage site of bradykinin.^3^ Loules G identified two variants as p.Pro574Ala (c.1720C>G) and p.Arg487Cys (c.1459C>T) in the year 2020 ^4^, followed by the identification of a variant of c.744T>A (pCys248∗]) in a Portuguese girl, which causes a cysteine substitution by a stop codon in the 248 position. Here, we found a hitherto novel variant with incomplete penetrance.

It is challenging to conclusively diagnose the inheritance pattern or the presence of incomplete penetrance at this stage from the limited data. In a previous report of Loules *G*, the variant p.Pro574Ala of the KNG1 gene also presents with incomplete penetrance, or its possible pathogenicity depends upon its compound heterozygosity with the ACE p.Arg487Cys variant.^4^ This suggests that variants in this gene can exhibit incomplete penetrance. However, these findings are based on different variants and may not directly apply to the specific variant we are studying. While the variants in *KNG1* suggest a possible autosomal dominant inheritance pattern, and the absence of clear clinical symptoms in III1 and III2 raises the possibility of incomplete penetrance, this remains speculative without further evidence.

Treatment of HAEnC1 depends on the clinical experience. Since the eight known pathogenic genes, including *KNG1*, are mainly correlated to the metabolism of bradykinin, icatibant is recommended as the on-demand treatment. Martinho et al reported the empirical treatment of icatibant in 16 cases of HAEnC1with significant efficacy.^5^ The KNG1 gene encodes kininogens, which are the source of bradykinin after cleavage. Icatibant demonstrated a fast and effective response at the empirical administration in our case. The subsequent follow-up reported another two experiences of successful on-demand administration of icatibant, which further confirmed the effect of icatibant in this patient.

Our study reported the first case of HAEnC1 due to *KNG1* mutation in China, and showed the powerful efficacy of icatibant in the acute attack of HAEnC1 empirically. It added the clinical experience of the on-demand treatment of HAEnC1. Further clinical trials are needed for the wide application.

## CRediT authorship contribution statement

**Wo Yao:** Investigation, Data curation. **Boyun Yang:** Validation, Methodology. **Huiying Wang:** Writing – review & editing, Writing – original draft, Methodology.

## Ethics declaration

This study was conducted in accordance with the Declaration of Helsinki and was approved by the Ethics Committee of the Second Affiliated Hospital of Zhejiang University School of Medicine (No. 2024-0014). No human experimentation is involved in this letter.

## Conflict of interests

The authors declare that they have no known competing financial interests or personal relationships that could have appeared to influence the work reported in this paper.
