# Supplementary material for: On-demand treatment of icatibant in a patient of hereditary angioedema with KNG1 mutation
Source: Genes Dis. 2026 May 14;14(1):102233. doi: 10.1016/j.gendis.2026.102233 (PMC13506498; doi:10.1016/j.gendis.2026.102233)
Supplement: Supplementary file 1 [file mmc1.docx]

**Supplementary data**

**Methods**

**Measurement of C4 and C1INH antigen and function**

C4 level was quantified using immunoturbidimetry by Beckman coulter AU5800 System (Siemens) . C1INH level was quantified using nephelometry by Atellica NEPH 630 System (Siemens) with N Antiserum to Human C1-Inhibitor as the reagent C1INH functional activity in the plasma was measured using Enzyme-Linked Immunosorbent Assay (ELISA) by MicroVue C1-Inhibitor Plus EIA by Quidel. Both tests were performed in Kingmed Center，Guangzhou. Results were normalized as percentage of normal value (C4 normal range 0.1−0.4 g/L; functional C1INH normal range ≥68%; C1INH level normal range 0.21-0.39 g/L).

**Whole Exome Sequencing and Analysis**

Genomic DNA of the patient and his family members was extracted according to the manufacturer's standard procedure using the QIAamp DNA Blood Midi Kit (Qiagen, Hilden, Germany). Then the genomic DNA of the family was fragmented by Covaris LE220 (Massachusetts, USA) to generate paired-end library (200-250 bp). The library was enriched by array hybridization according to manufacturer's standard procedure, followed by elution and post-capture amplification. The products were then subjected to Agilent 2100 Bioanalyzer and ABI StepOne to estimate the magnitude of enrichment. After quality control, captured library sequencing was carried out on Illumina HiSeq X Ten Analyzers (Illumina, SanDiego, USA. Following the manufacturer's standard sequencing protocols) for 150 cycles per read to generate paired-end reads. Image analysis, error estimation, and base calling were performed using Illumina Pipeline software to generate raw data.

To detect the potential variants, we performed bioinformatics processing and data analysis after receiving the primary sequencing data. We using AfterQC to generate “clean reads” for further analysis. The “clean reads” (with a length of 150 bp) derived from targeted sequencing and filtering were then aligned to the human genome reference (hg19) using the BWA (Burrows Wheeler Aligner) software. After alignment, the output files were used to perform sequencing coverage and depth. We used GATK (Genome Analysis Toolkit) software to detect SNVs and indels. All SNVs and indels were filtered and estimated via multiple databases, including 1000 Genomes (1000 human genome dataset), Genome AD (Genome Aggregation Database dataset) and ExAC (The Exome Aggregation Consortium dataset). We used dbNSFP to predict the effect of missense variants. The Human Gene Mutation Database (HGMD) and Clinvar Database was used to screen mutations reported in published studies. Pathogenic variants are assessed under the protocol issued by ACMG.

**Protein modeling and functional prediction**

The pathogenic missense mutation KNG1 c.1483C>G (p.His495Asp) was analyzed using the reference transcript NM_001102416.3 (UniProt ID: P01042). The mutation substitutes histidine (His) at position 495 with aspartic acid (Asp), altering residue properties from basic to acidic. Genomic coordinates (chr3:186,717,359-186,744,410) and domain annotation (InterPro, UniProt) confirmed residue 495 lies outside functional domains, including the cystatin-like domain (residues 266–370). Secondary structural changes were evaluated using SOPMA (https://npsa-prabi.ibcp.fr). Analysis indicated alterations in the proportions of α-helices (Hh), β-turns (Tt), and random coils (Cc), while extended strands (Ec), 310-helices (Gg), and other elements remained unchanged. This suggests potential disruption of local secondary structure. Structure prediction with wild-type (WT) and mutant (His495Asp) protein structures were modeled using AlphaFold2 (https://colab.research.google.com/github/sokrypton/ColabFold) analyzed with PyMOL v2.0, which visualized no hydrogen bond alterations at residue 495, implying preserved backbone conformation. Electrostatic potential were calculated with ChimeraX v1.6 and the APBS tool, which revealed a shift from neutral (WT) to negative surface charge at residue 495, indicating disrupted electrostatic interactions.
